# Visualization of small vibrations inside an MRI scanner using video motion amplification

**DOI:** 10.1002/mp.70315

**Published:** 2026-02-26

**Authors:** Youngseob Seo, Zhiyue J. Wang

**Affiliations:** ^1^ Group for Emerging Research Instruments Korean Research Institute of Science and Standard Daejeon Republic of Korea; ^2^ Department of Radiology University of Texas Southwestern Medical Center Dallas Texas USA

**Keywords:** diffusion‐weighted MRI, high‐speed video, motion amplification, quality assurance, visualization of vibration

## Abstract

**Background:**

Diffusion‐weighted magnetic resonance imaging (DW‐MRI) acquisition requires the application of strong magnetic field gradients, which can induce mechanical vibrations in tissues or phantoms, potentially leading to signal loss or degradation. A qualitative assessment of these vibrations would be valuable for quality assurance (QA). Conventional methods, such as piezoelectric accelerometers and laser interferometry, have limitations in their applicability and availability. There remains a need for a readily accessible method to detect and characterize these vibrations as part of a robust QA protocol.

**Purpose:**

The objective of this study is to investigate the feasibility of using motion amplification of high‐speed video to assess vibrations during DW‐MRI scanning.

**Methods:**

A gel phantom simulating a human head was positioned supine within a head receive coil inside an MRI scanner. A 45‐degree angled mirror was placed to visualize the phantom's face, while a high‐speed camera, positioned outside the scanner, was used to record videos under two conditions: (1) the scanner in an idle state (still condition), and (2) during a DW‐MRI scan (vibration condition). The recorded videos were processed using a motion amplification software tool to enhance subtle movements. The motion of multiple position markers affixed to the phantom was quantitatively analyzed.

**Results:**

No motion was visible to the naked eye under either still or scanning conditions. However, motion amplification revealed clear marker displacement during DW‐MRI, with substantially smaller movement during scanner idling. Across all facial markers and directions (X (L‐R), Y (A‐P) and Z (I‐S)), median root‐mean‐square displacement increased from 0.85 µm (range: 0.61–1.50 µm) at idle to 2.59 µm (2.04–4.31 µm) during DW‐MRI scanning (b = 2500 s/mm^2^). Similarly, median peak‐to‐peak displacement rose from 7.71 µm (3.92–10.37 µm) to 18.47 µm (15.22–31.77 µm).

**Conclusions:**

Motion amplification of high‐speed video provides a viable method for detecting and analyzing vibrations during MRI scans. This approach could serve as a valuable tool for QA, offering an alternative to conventional vibration assessment techniques.

## 1 Introduction

Diffusion‐weighted magnetic resonance imaging (DW‐MRI) is a powerful imaging technique that has revolutionized the field of MR imaging by providing detailed insights into tissue microstructure.^1^, ^2^, ^3^, ^4^, ^5^, ^6^, ^7^, ^8^, ^9^, ^10^, ^11^, ^12^, ^13^, ^14^, ^15^, ^16^ Unlike conventional MRI, which primarily visualizes anatomical structures, DW‐MRI captures the diffusion of water molecules within tissues, offering critical information about tissue architecture and cellular integrity. This makes it particularly valuable in the diagnosis and assessment of neurological disorders, such as stroke, multiple sclerosis, and brain tumors, where changes in the diffusion of water can indicate early pathological alterations.^17^, ^18^, ^19^, ^20^, ^21^, ^22^, ^23^, ^24^, ^25^, ^26^, ^27^, ^28^, ^29^, ^30^, ^31^, ^32^ Moreover, diffusion tensor imaging (DTI), a specialized form of DW‐MRI, allows for the mapping of white matter tracts in the brain, aiding in both surgical planning and the study of neurodegenerative diseases.^33^, ^34^, ^35^, ^36^, ^37^ The non‐invasive nature of DW‐MRI, combined with its ability to detect subtle changes in tissue, has made it indispensable in clinical diagnostics and research, particularly in the fields of neurology and oncology. However, DW‐MRI requires the application of strong magnetic field gradients during the imaging sequence to encode the diffusion of water molecules in tissue, which may induce mechanical vibrations in both the MRI hardware and the subject. These vibrations affect image quality and signal integrity, underscoring the need for reliable quality assurance (QA) measures.^38^, ^39^, ^40^, ^41^, ^42^, ^43^ These vibrations are more pronounced in certain areas of the body, leading to artifacts such as signal loss and errors in quantitative assessment of diffusion metrics.

One common artifact resulting from these vibrations is the loss of signal in areas where vibration‐induced tissue motion aligns with the diffusion‐encoding gradient direction. This effect can be exacerbated in patients with low body mass, such as neonates, due to reduced mechanical damping.^44^ In such cases, increased tissue mobility amplifies the extent of motion artifacts, resulting in inaccurate depiction of diffusion anisotropy and potentially impairing diagnostic accuracy. These artifacts necessitate careful monitoring and implementation of QA techniques to ensure accurate imaging, particularly in sensitive clinical applications like neurology or oncology.

Despite the critical importance of maintaining image quality in DW‐MRI, there is a notable lack of standardized QA methods to evaluate the MRI‐induced vibration, particularly in diffusion‐weighted imaging (DWI). Traditional QA protocols primarily focus on parameters such as field homogeneity, signal‐to‐noise ratio (SNR), and geometric accuracy, with little attention paid to detecting and quantifying vibration‐induced artifacts. Implementing QA procedures would allow us to ensure that there is no excessive vibration during the scan, as well as to evaluate the effectiveness of measures taken to reduce the vibrations.

Current methods for assessing vibration, such as piezoelectric accelerometers and laser interferometry, present practical limitations within the MRI environment. Commercial piezoelectric accelerometers require probe mounting and are generally incompatible with the strong magnetic fields of MRI systems. Laser interferometry is also limited to detect motion at a single point and in a single direction, meaning motion perpendicular to the light beam cannot be detected. This directional constraint makes it challenging to capture the full scope of vibrational effects, especially in regions where multidirectional tissue motion is likely. In addition, the laser interferometry requires expensive equipment which is often not affordable due to budget constraints. The lack of a comprehensive and easy‐to‐implement QA method allows vibration issues to go undetected, potentially compromising the accuracy of both clinical diagnoses and research findings.

To address these limitations, alternative non‐contact and MRI‐compatible techniques are required. Motion amplification of video has emerged as a promising tool for vibration assessment, overcoming the limitations of conventional methods like piezoelectric accelerometers and laser interferometers.^45^, ^46^, ^47^, ^48^ By capturing high‐speed video and algorithmically enhancing subtle, otherwise imperceptible motion, this technique enables the visualization of vibration across the entire field of view. Importantly, it operates independently of the magnetic environment and can be integrated into existing clinical workflows with minimal disruption. Motion amplification provides spatially enough information on vibrational dynamics, thereby supporting protocol optimization and enhancing image quality and diagnostic reliability.

In this study, we investigate the feasibility of using motion‐amplified video recordings to assess vibration during diffusion MRI acquisition in a phantom. The proposed approach is evaluated as a potential QA tool for identifying and characterizing vibration that may compromise image fidelity.

## 2 Materials and Methods

### 2.1 Phantom construction and setup

**FIGURE 1:**
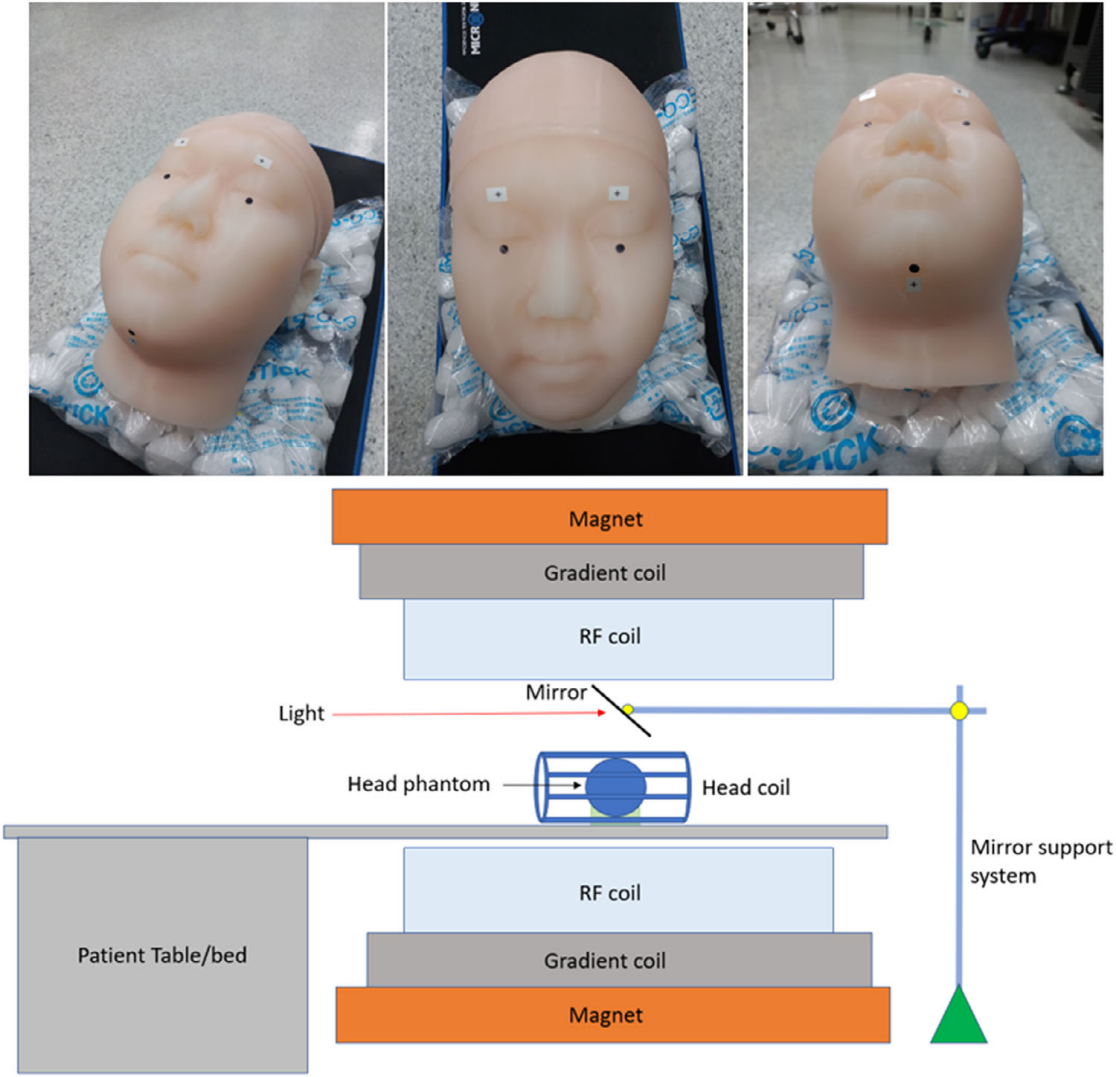
Photographs of a gel phantom simulating a human head with markers (top) and a schematic diagram showing a mirror placed above the phantom at a 45‐degree angle (bottom).

A human head phantom corresponding to an adult male subject was constructed for this study (Figure 1). The dimensions of the head phantom were as follows: a head width of 15.8 cm, a head circumference of 56.8 cm, and a vertical head length of 22.7 cm. The outer shell of the head phantom was fabricated using 1.3 kg of Ecoflex™ 00–50 silicone rubber (Smooth‐On Inc., Macungie, PA, USA), which is softer than human skin. The interior cavity of the phantom was filled with 4.0 kg of saline solution, consisting of distilled water mixed with 0.9% NaCl by weight to mimic the human brain.^49^ The phantom was positioned within a standard MRI head receive coil inside a 3T Magnetom Verio MRI scanner (Siemens Healthcare, Germany), placed in the supine position to replicate typical clinical conditions. To facilitate video capture, a mirror was positioned above the phantom at a 45‐degree angle, providing an unobstructed view of the phantom's face. This setup allowed the high‐speed camera to capture subtle motion of the phantom during the MRI scanning process.

### 2.2 Video acquisition

The DW‐MRI acquisition was performed using a single‐shot echo‐planar imaging (EPI) sequence with the following parameters: field of view (FoV) = 256 × 256 mm^2^, matrix size = 256 × 256, voxel size = 1 × 1 × 2 mm^3^, repetition time (TR) = 9697 ms, echo time (TE) = 83 ms, *b* = 2500 s/mm^2^, gradient strength = 40 mT/m, number of slices = 62, slice thickness = 2 mm, number of averages = 2, and diffusion encoding directions = 3. These settings were chosen to replicate typical DWI protocols, which involve strong magnetic field gradients known to induce mechanical vibrations. Sound recordings were collected to determine the timing of each encoding gradient direction. The acquisition time was 87 s. The sound changed approximately at 12, 37, and 62 s. Thus, the MRI acquisition time was divided as follows: *b* = 0: 0–12 s; first gradient direction: 12–37 s; second gradient direction: 37–62 s; and third gradient direction: 62–87 s.

A high‐speed camera (Photron Limited FASTCAM Mini WX100, Tokyo, Japan) was positioned on a tripod outside the MRI bore and aimed at the head phantom and the mirror to capture the phantom's face via its reflection. The accuracy of the frame rate is ± 50 ppm according to specification. The camera recorded video at a frame rate of 4000 frames per second (fps) with a resolution of 1280 × 1024, allowing for the detection of subtle vibrations or motions. Two video acquisition conditions were used: (1) a baseline or still condition, where the MRI scanner was idle and not performing any scans, and (2) a scanning condition, during which a DW‐MRI sequence was applied. The total video recording time was 4.4 s, and videos were recorded for each of the four different time slots.

### 2.3 Motion amplification processing

Motion amplification in video is a technique that enhances subtle movements in a video sequence, making otherwise imperceptible motion visible to the naked eye. It operates by processing each frame of a high‐speed video to identify and amplify motion‐related pixel intensity variations at specific frequencies. Using algorithms such as Eulerian Video Magnification (EVM),^46^, ^50^, ^51^ the technique isolates small changes in pixel intensity corresponding to motion and increases their amplitude. For reproducibility, the EVM implementation used in this study was based on the publicly available open‐source framework described in the previous studies.^46^, ^48^ The software is publicly available at https://people.csail.mit.edu/mrub/evm/#code. This approach enables detailed visualization of vibrations, deformations, or other subtle movements that are too small to detect through normal observation, rendering the technique valuable for applications such as vibration analysis in an MRI environment.

In this study, following video acquisition, the EVM technique was applied to selectively amplify subtle movements within a specified frequency band, making even imperceptible vibrations visible. The frequency band associated with MRI‐induced vibrations was identified and amplified to assess the motion of the head phantom during both idle and scanning conditions. Amplification factors were adjusted iteratively until the smallest detectable movement was clearly visible. The parameters used for the motion amplification were: frequency band from 10 to 1000 Hz, an amplification factor of 100. This frequency range was selected because it corresponds to expected gradient‐induced mechanical resonances of scanner hardware components (coil, boom, table), as reported in the previous work,^42^ and was adjusted based on the resulting peak distribution in the frequency domain. The amplification factor was chosen empirically to ensure clear visibility of vibration patterns without introducing saturation or excessive motion artifacts.

### 2.4 Data analysis

All data analyses were performed using custom‐written Python scripts. Motion in both the still and scanning conditions was evaluated by tracking fiducial markers placed on the surface of the phantom. These markers allowed for precise measurement of displacement over time. In each motion‐amplified video segment, the movements of four markers were tracked, including those on the right forehead, the left forehead through the mirror, and the nose and chin in direct view. The chin and nose markers exhibited motion primarily in the X (L‐R) and Y (A‐P) directions within the transverse plane, while the forehead markers reflected in the mirror showed motion mainly in the Z (I‐S) directions. Displacement data were analyzed to calculate the frame‐to‐frame position of the center of each cross‐shaped marker. Statistical analysis was performed to determine the significance of the observed movements during the DW‐MRI scans compared to the baseline still condition.

To determine the pixel size in the video image, a cross‐shaped marker was used. The marker size (horizontal and vertical lengths) was first measured using a microscope (Model VHX‐7020, Keyence Korea Ltd., Seoul, South Korea) and a stage micrometer with 0.001 mm precision (Model PS12, Graticules Optics Ltd., Cambridge, United Kingdom). Then, in the high‐speed camera images, the corresponding number of pixels covering the same horizontal and vertical marker lengths was counted. The pixel size (mm/pixel) for each direction was obtained by dividing the known physical length of the marker by the number of pixels. The pixel resolution of the processed video corresponded to 0.49 ± 0.01 mm/pixel along the X (L‐R) axis (horizontal) and 0.46 ± 0.01 mm/pixel along the Y (A‐P) axis (vertical) within the image FoV. For spectral analysis, Fourier transformation was applied separately to the X (L‐R) and Y(A‐P) components of the displacement, which were subsequently combined to derive the amplitude spectrum.

Multiple repeats per condition were not acquired in this initial feasibility study. However, to assess intra‐run stability, each video clip was divided into two halves, and the root‐mean‐square (RMS) values of the first and second segments were calculated and compared using the Bland–Altman analysis.^52^ The percent difference between the two segments was quantified as the mean difference and the 95% confidence intervals for the limits of agreement.

To evaluate differences between idle and scanning conditions, bootstrap resampling and permutation *t*‐tests were performed for each marker and motion direction to determine statistical significance.

## 3 Results

**FIGURE 2:**
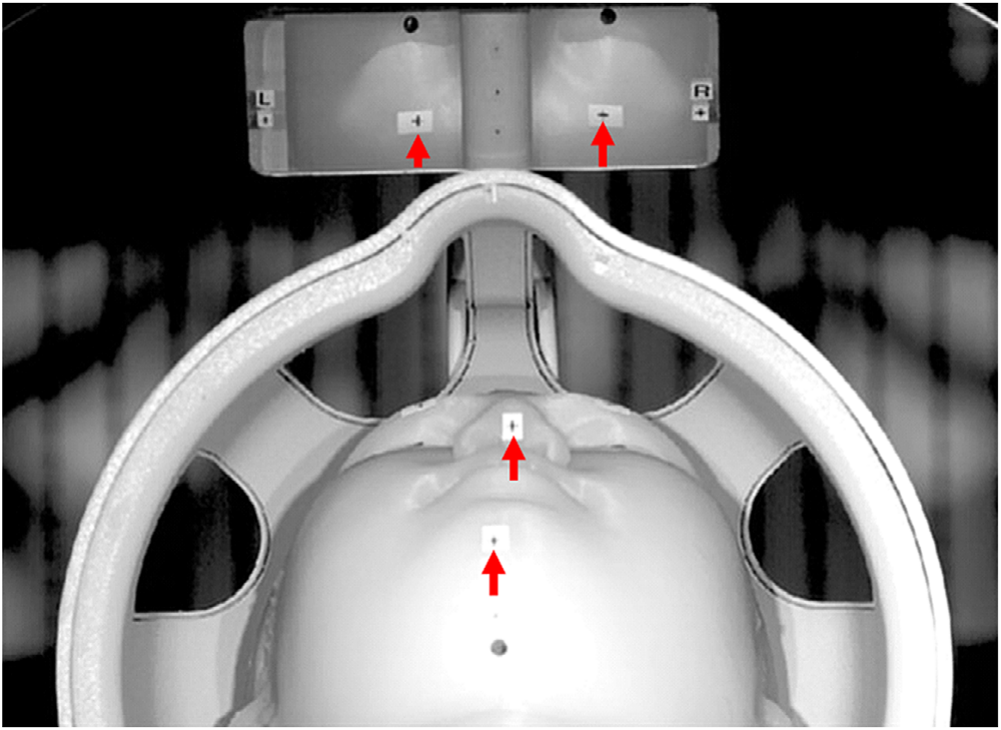
One video frame showing the processed video field of view. Red arrows point to the markers used for displacement quantification. The matrix size of the image is 619 × 459 pixels. See Supporting Video S1.

Figure 2 shows one frame of the processed video. None of the videos exhibited visible motion when viewed with the naked eye without motion amplification. Supplementary video S1 provides a comparison between the raw and processed videos, with the left panel displaying the unamplified video and the right panel showing the same sequence after motion amplification. In the motion‐amplified videos, the phantom motion was observed during the DWI scans, but it was much smaller in the still condition. Different diffusion encoding directions gave rise to distinct vibration patterns. When the diffusion encoding gradient switched directions, a small jump occurred in the position of both the radio‐frequency (RF) coil and the phantom.

Table 1 summarizes the motion statistics for four marker positions under still conditions and three diffusion encoding directions. Marker displacements were quantified across all facial landmarks (forehead, nose, and chin) and all spatial directions (X (L‐R), Y (A‐P) and Z (I‐S)), and then converted to physical units using pixel dimensions of 0.49 mm (X) and 0.46 mm (Y). In Table 1, ΔX, ΔY, and ΔZ denote displacement along the indicated axis relative to each marker's mean position in the motion‐amplified video segment. Each marker was tracked in two in‐plane directions (either X and Y or X and Z, depending on the marker). The summary statistics pool all measured marker‐direction values within each condition listed in Table 1. The reported medians and ranges therefore reflect the distribution across all marker‐direction values (two per marker), regardless of whether they correspond to X/Y or X/Z. During scanner idling, the median RMS displacement was 0.85 µm (range: 0.61–1.50 µm), and the median peak‐to‐peak (p‐p) displacement was 7.71 µm (range: 3.92–10.37 µm). During acquisition of *b* = 0 diffusion images, the median RMS displacement increased to 1.38 µm (range: 1.25–1.59 µm), with a median p‐p displacement of 9.52 µm (range: 8.56–12.17 µm). In contrast, DW‐MRI acquisition with b = 2500 s/mm^2^ produced a median RMS displacement of 2.59 µm (range: 2.04–4.31 µm) and a median p‐p displacement of 18.47 µm (range: 15.22–31.77 µm). These values reflect a consistent increase in facial marker motion during scanning, with the largest displacements observed near the chin and left forehead. Baseline motions were present in the still condition, possibly due to low levels of inherent motion and video noise. In addition, the two forehead markers viewed via the mirror exhibited larger baseline motions than the marker in direct view.

**TABLE 1:** Root‐mean‐square (RMS) and peak‐to‐peak (p‐p) marker displacements (µm) during scanner idling and diffusion‐weighted acquisitions, computed relative to each marker's mean position within the corresponding video segment. Values are reported per marker and tracked image‐plane direction (forehead: X–Z; nose/chin: X–Y). Diffusion directions 1–3 correspond to encoding axes X (L–R), Y (A–P), and Z (I–S), respectively.

	Right forehead				Left forehead			
	ΔX		ΔZ		ΔX		ΔZ	
	RMS	p‐p	RMS	p‐p	RMS	p‐p	RMS	p‐p
Scanner idling	0.81	7.14	1.13	8.29	0.85	9.90	1.47	10.37
Diffusion *b* = 0	1.27	9.07	1.39	9.27	1.52	12.17	1.59	11.75
Diffusion direction 1	2.32	18.07	2.24	17.24	3.16	26.03	2.82	19.34
Diffusion direction 2	2.50	17.98	2.24	15.22	3.05	21.60	2.92	22.66
Diffusion direction 3	2.79	18.87	2.43	17.30	3.17	24.50	3.34	20.54

	Nose				Chin			
	ΔX		ΔY		ΔX		ΔY	
	RMS	p‐p	RMS	p‐p	RMS	p‐p	RMS	p‐p
Scanner idling	0.61	3.92	0.84	6.36	0.80	5.71	1.50	9.71
Diffusion *b* = 0	1.40	9.79	1.25	8.85	1.38	11.47	1.32	8.56
Diffusion direction 1	2.29	17.76	2.50	17.48	2.62	17.27	2.56	16.49
Diffusion direction 2	2.04	16.83	4.31	21.31	2.50	17.98	2.24	15.22
Diffusion direction 3	2.47	19.84	3.37	18.92	3.09	29.56	3.66	31.77

**FIGURE 3:**
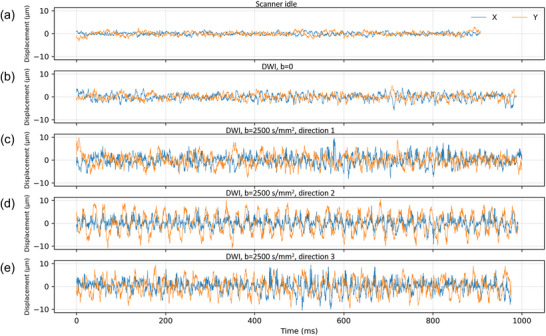
Time domain trace of the nose marker motion in the X (L‐R) and Y (A‐P) plane across imaging conditions. (A) Scanner idle (still condition); (B) Diffusion‐weighted imaging with *b* = 0; (C) Diffusion encoding direction 1 (X‐direction); (D) Diffusion encoding direction 2 (Y‐direction); and (E) Diffusion encoding direction 3 (Z‐direction). The vertical scale is the same for all conditions for direct comparison. Acquiring DWI *b* = 0 images increased vibration relative to the idle condition, and vibration increased further during b = 2500 s/mm^2^ acquisitions.

The results of the intra‐run RMS stability analysis are summarized in Table 2. The assumption of a true steady‐state condition is only approximate. In addition, the video recording was not synchronized with the MRI pulse sequence. During each recording, images from multiple slices were acquired, and the exact relative timing between the pulse sequence and the video was not tracked. Given these limitations, the level of the agreement observed in Table 2 appears reasonable. The intra‐run stability is also evident in Figure 3, as illustrated for the nose marker.

**TABLE 2:** The Bland–Altman analysis of agreement between the first‐ and second‐half movement RMS values (1st–2nd). All markers and motion directions were pooled within each condition (*n* indicates the number of paired first‐ and second‐half measurements). For *b* = 2500 s/mm^2^, data from all diffusion‐encoding directions were combined.

Condition	Mean difference (%)	95% confidence intervals for limits of agreement (%)
Idle (*n* = 8)	−2.7	−15.3 to 10.0
Diffusion *b* = 0 (*n* = 8)	−7.0	−14.2 to 0.3
Diffusion *b* = 2500 s/mm^2^ (*n* = 24)	−4.9	−30.9 to 21.1

In all markers and spatial directions (X (L‐R), Y (A‐P), and Z (I‐S)), the 95% bootstrap confidence intervals for RMS displacement differences excluded zero, and the permutation tests yielded two‐sided *p* < 0.001, indicating that the idle condition differed significantly from all scanning conditions.

**FIGURE 4:**
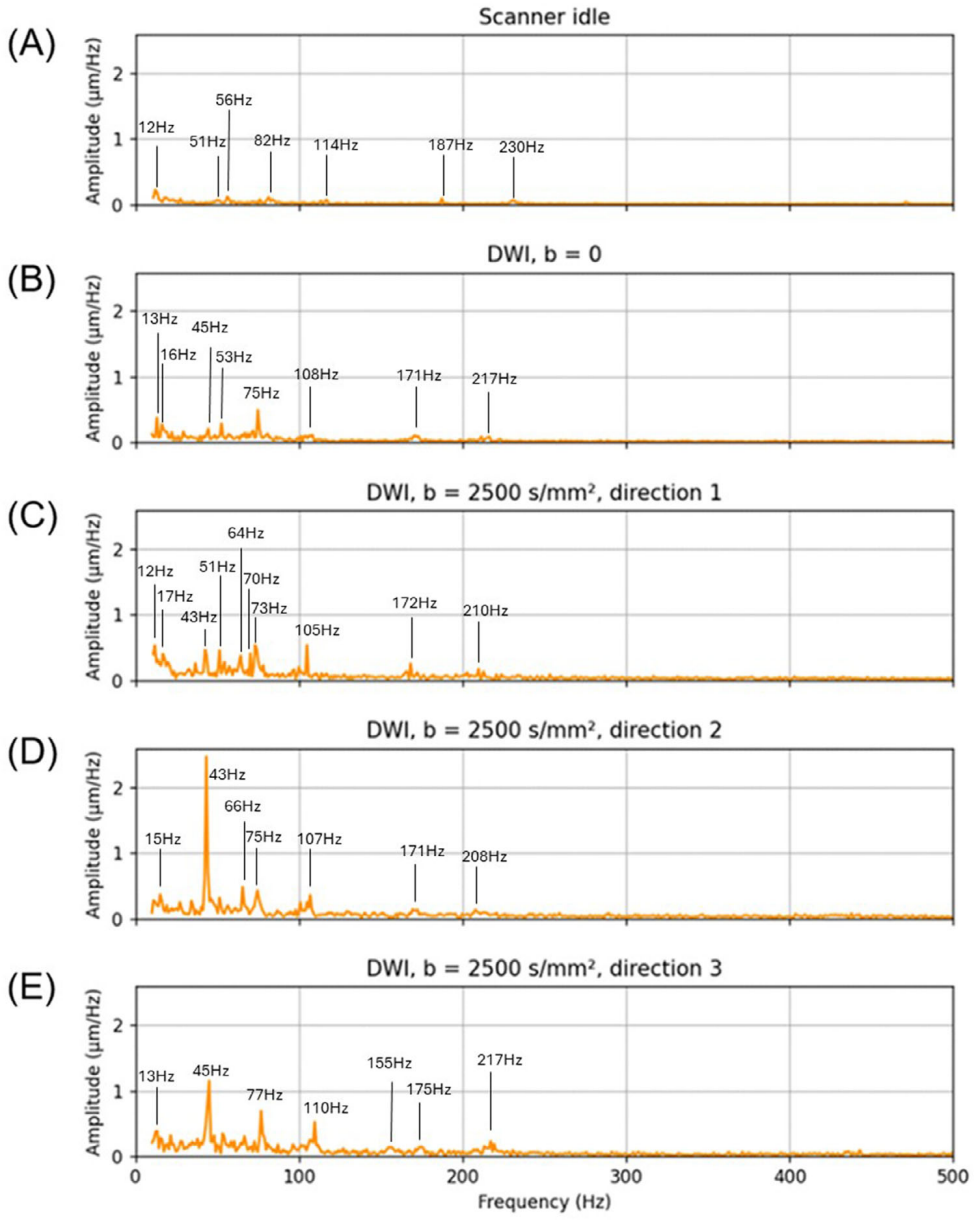
Amplitude spectrum of nose marker motion in the X (L‐R) and Y (A‐P) plane across imaging conditions. (A) Scanner idle (still condition); (B) Diffusion‐weighted imaging with *b* = 0; (C) Diffusion encoding direction 1: X (L‐R); (D) Diffusion encoding direction 2: Y (A‐P); and (E) Diffusion encoding direction 3: Z (I‐S). The vertical axis shows motion amplitude in micrometer per Hertz (µm /Hz), reflecting the estimated physical motion before motion amplification. The results for the bandwidth of 500 to 1000 Hz are not shown because this frequency range contains minimal motion.

Figure 4 shows the motion amplitude spectrum of the nose marker under the five conditions. Under the scanner idle condition (Figure 4A), minimal motion amplitude was observed, with maximum low‐level broadband components below 20 Hz. In contrast, the DWI *b* = 0 condition (Figure 4B) introduced a noticeable increase in the vibration amplitude, particularly in the frequency band below 110 Hz. As diffusion encoding gradients were applied (Figure 4C‐E), vibration amplitudes increased. Diffusion encoding direction 1 (Figure 4C) exhibited a moderate rise in spectral power, while encoding direction 3 (Figure 4E) showed higher peaks in the frequency band below 110 Hz than direction 1. The most pronounced vibration occurred with encoding direction 2 (Figure 4D) in the frequency range around 30–60 Hz. The spectrum contains multiple sharp frequency components, similar to those observed in laser interference vibration measurements during MRI scans.^53^, ^54^

## 4 Discussion

In current medical physics practice, QA measurements for vibration assessment during MRI scans are lacking. This study represents the first demonstration of using video motion amplification to visualize and quantify subtle vibrations during diffusion MRI scans. A previous work has validated the vibration measured by the video amplification approach.^47^

Unlike conventional QA metrics in diffusion MRI, such as SNR and geometry fidelity, our approach directly addresses the often‐overlooked issue of mechanical vibration. The frequency‐domain analysis presented here revealed distinct spectral fingerprints corresponding to each diffusion encoding direction, which are not captured by traditional QA methods. This underscores the added diagnostic value of motion‐amplified video analysis as a complementary QA tool.

Traditional methods such as piezoelectric accelerometers and laser interferometry can only detect displacement in one direction with a single probe. In contrast, the video measurement approach enables two‐dimensional motion detection in a single plane simultaneously, providing a more complete assessment of vibrational dynamics. Moreover, this technique is non‐contact and MRI‐compatible, allowing integration into existing scanning workflows without interference.

To obtain reliable motion quantification, adequate lighting conditions are important. Dim lighting can result in increased noise that may be misinterpreted as displacement during motion amplification processing. Furthermore, the use of a mirror appears to introduce artifactual motions, suggesting the need for technical improvements to minimize the movement of the mirror.

In this study, a frequency bandpass range of 10–1000 Hz was applied during motion amplification for demonstration purposes. Figure 2 displays the resulting motion amplitude spectrum up to 500 Hz. Higher frequencies are not shown, as they contain minimal motion—likely due to increased damping by the silicone rubber phantom surface at those frequencies. The dominant vibration amplitudes fall within this displayed range, although higher frequency components are present. The high frame rate of our video recordings (4000 fps) supports analysis well beyond 500 Hz if needed; however, based on the observed vibration spectra, high‐speed cameras operating in the 500–1000 fps would likely suffice for most practical QA applications. The use of 4000 fps videos in this feasibility study was intended to maximize temporal resolution and ensure that no higher‐frequency components were missed. In addition, using a lower frame rate may allow longer video recording times and less noise, which will be beneficial.

Quantitative analysis (Table 1) revealed subpixel displacements ranging from 0.61 to 4.31 µm (RMS) and from 3.92 to 31.77 µm (p‐p) under both still and scanning conditions. These small‐scale vibrations, which are not detectable by the naked eye or standard QA tools, were reliably quantified for direction‐specific diffusion encoding. It is important to note that the extent of vibration is expected to be phantom‐dependent—resonance between the phantom and the scanner table may enhance or dampen motion at certain frequencies, underscoring the importance of system‐specific evaluation in QA assessment. Forehead markers showed slightly higher baseline motion, likely due to mirror instability. Future studies could use a stiffer mirror mount or an alternative direct‐view approach to mitigate this mirror artifact. The current phantom setup enables repeated testing of the nose and chin markers, which are directly visible, without using a mirror. Using a mirror to image the forehead markers may introduce additional variability across repeated tests.

While these submicrometer‐scale displacements could be quantified with the proposed approach, it is important to consider the effective noise floor, detection limit, and reproducibility of the measurements. In the present experiments, the smallest measured displacements occurred under idle conditions, with RMS values on the order of 0.61–0.85 µm (Table 1). Although low‐level motion from sources such building vibration could not be ruled out, this range likely reflects the lower bound of reliably detectable motion given the current camera resolution, lighting conditions, and motion amplification pipeline, and may therefore be interpreted as an approximate detection floor of the system. Motions smaller than this level may be difficult to distinguish from video noise or residual tracking uncertainty. Explicitly stating this limitation is important for QA interpretation, as apparent low‐amplitude vibrations near this threshold should be treated with caution. With respect to reproducibility, the intra‐run Bland–Altman analysis demonstrated good stability of displacement estimates within a single acquisition session, with narrow limits of agreement indicating consistent measurements across repeated segments. However, inter‐run or session‐to‐session reproducibility was not evaluated in this feasibility study. Variability across days, scanner warm‐up states, or environmental conditions may influence absolute vibration amplitudes and spectral features. Future work will therefore include repeated full‐session measurements acquired on different days to quantify inter‐run reproducibility and to establish confidence intervals for long‐term QA monitoring. Such data will be essential for defining robust site‐specific thresholds and for distinguishing true system changes from normal measurement variability.

As shown in the supplementary video, the coil and phantom did not always move in phase. The rigid coil primarily reflects global vibrations transmitted through the scanner structure, whereas the soft silicone phantom exhibits additional local motion due to its compliant mechanical properties. These differing responses are consistent with resonance effects, in which the phantom's material properties and geometry influence motion at specific frequencies. The observed coil–phantom phase differences likely result from a combination of scanner‐induced vibration and phantom‐specific resonances. This observation has been noted to clarify the mechanical dynamics revealed by motion amplification. However, definitive conclusions would require quantitative analysis, which is beyond the scope of this preliminary study.

Importantly, synchronized audio recordings acquired alongside the video data could be leveraged for additional insight. In this study, however, the audio signals were used primarily to synchronize the video recording with the gradient encoding directions. Precise temporal alignment between audio and video was not performed, and the audio was recorded with suboptimal quality, limiting its value for quantitative analysis. In principle, frequency‐domain comparison of audio and motion signals could confirm that the observed motions are driven by scanner vibrations and reveal additional harmonics or resonance phenomena within the magnet. Correlating audio and motion spectra remains a promising avenue for future work and supports the use of multimodal (video + audio) recordings as a complementary tool for comprehensive QA assessment.

In MRI environments that use eye tracking, near‐infrared (NIR) light sources, invisible to the subject, are often employed to illuminate the head. High‐speed cameras capable of detecting such NIR illumination are commercially available and commonly used in biomechanics and machine vision. Integration of an NIR‐based illumination system with high‐speed video acquisition could improve practicality by avoiding visible light exposure and ensuring robust marker detection. Although our current setup relied on visible illumination, adopting NIR‐compatible high‐speed cameras could facilitate translation to clinical studies, particularly for patient populations.

The vibration spectrum of marker motion across imaging conditions revealed that DWI, particularly with higher *b*‐values and specific gradient encoding directions, induces substantial mechanical vibrations in the scanning environment. The idle condition confirms a low baseline of scanner‐induced vibration, while the introduction of imaging gradients significantly increases marker motion. This suggests that rapid gradient switching can be key contributors to system vibrations.

It is well known that mechanically induced micro‐motions during diffusion encoding could introduce subtle distortions or errors in apparent diffusion coefficient (ADC) estimation.^40^, ^44^ Transient micro‐displacements may lead to intravoxel dephasing or phase incoherence across gradient pulses, thereby altering signal attenuation beyond the purely diffusion‐driven effect. Such distortions could, in principle, cause bias in ADC values (either overestimation or underestimation, depending on the phase accumulation) or contribute to localized signal dropout, particularly in EPI sequences with long readouts. Indeed, prior reviews note that diffusion MRI is highly sensitive to motion and hardware imperfections, and that gradient switching and eddy currents remain important sources of artifacts in DWI acquisitions.^41^, ^43^, ^55^, ^56^ Future studies are needed to extend this analysis beyond ADC and to quantify changes in DTI metrics (e.g., fractional anisotropy, mean diffusivity and axial diffusivity), as well as to assess how these changes correlate with observed surface vibrations.

From a clinical QA perspective, even a conceptual estimate of the magnitude of such bias is valuable. For instance, if the amplitude of micro‐motion corresponds to a non‐negligible fraction of voxel dimensions (say a few micrometers), the resulting phase errors during diffusion encoding could yield percent‐level perturbations in ADC maps. While this is speculative, it provides a bridge from our mechanistic measurements to imaging outcomes. In applied settings, one might consider phantom experiments or motion‐sensitive calibration scans to quantify the residual bias in scanner systems. Embedding such checks into QA protocols would help ensure that direction‐dependent vibration modes or mechanical resonances do not silently degrade diffusion measurement reliability in routine clinical scans.

Although this study used a head phantom, these findings have important implications for clinical imaging, particularly in sensitive populations such as neonates and other pediatric patients. In these groups, increased tissue compliance and lower body mass may exacerbate vibration effects, potentially leading to greater motion artifacts. Early detection through this non‐contact QA tool could enable clinicians to proactively adjust gradient settings or patient positioning to mitigate risk.

This proposed method can be implemented without interfering with the MRI scanning protocol, using a simple high‐speed camera setup external to the magnet bore. Its compatibility with clinical environments and relatively low cost make it a viable candidate for integration into routine QA practices, especially in high throughput neuroimaging facilities.

Resonance frequencies and vibration amplitudes vary across phantoms and MRI systems; therefore, each site could use our high‐speed video motion amplification method to establish local baselines under standard scanning conditions, capturing typical vibration patterns for that specific system. The same method can then be applied following hardware service, gradient coil maintenance, or sequence updates to monitor changes relative to the baseline. This method is non‐invasive, requires minimal equipment requirements, and provides quantitative displacement measurements, making it suitable for both longitudinal QA monitoring and the establishment of site‐specific thresholds.

To address practical QA implementation, a feasible pathway is to involve routine phantom‐based monitoring. Specifically, quarterly DW‐EPI phantom scans are to be acquired with a small set of short video clips (e.g., 3–5 acquisitions), from which quantitative vibration metrics such as RMS displacement, p‐p amplitude, and dominant frequency peaks are reported relative to a predefined baseline tolerance. For scanners that frequently image low–body weight patients or exhibit vibration artifacts in diffusion imaging, sites may implement more frequent QA intervals (e.g., monthly) at their discretion. This framework is intended to provide a systematic method for detecting scanner‐specific drifts in vibrational behavior over time and for establishing actionable thresholds that can trigger maintenance or recalibration. Importantly, by embedding such measurements within a scheduled QA protocol, the feasibility demonstrated here can be translated into a reproducible practice for clinical and research MRI facilities.

Future studies will focus on refining this technique, optimizing it for clinical settings, exploring its application in human studies, and performing direct validation with synchronized multimodal recordings (video and audio) to investigate potential audio‐motion correlations and strengthen QA confidence. Repeated measurements need to be included in future studies to further enhance reproducibility. These advancements could contribute to ensuring high‐quality and reliable diffusion MRI in both clinical examinations and research.

## 5 Conclusions

This study demonstrates the feasibility of using motion‐amplified video analysis to assess vibration induced during diffusion MRI sequences. By capturing subtle motions of a head phantom with sub‐millimeter sensitivity, this method reveals encoding direction‐specific vibration patterns that are otherwise imperceptible. The frequency spectra and displacement statistics highlight the capacity of this technique to detect potentially image‐degrading vibrations. Given its non‐contact nature, compatibility with MRI environments, and ability to analyze multidirectional motion, this approach offers a promising addition to QA protocols in both research and clinical diffusion MRI.

## CONFLICT OF INTEREST STATEMENT

The authors declare no conflicts of interest.

## Supporting information



Supplementary video S1. Comparison of raw and processed videos. The left panel shows the unamplified video, and the right panel shows the same sequence after motion amplification.

## Data Availability

Experimental data from this study are available to interested readers upon reasonable request.
